# Association of food security status with overweight and dietary intake: exploration of White British and Pakistani-origin families in the Born in Bradford cohort

**DOI:** 10.1186/s12937-018-0349-7

**Published:** 2018-04-24

**Authors:** T. C. Yang, P. Sahota, K. E. Pickett, M. Bryant

**Affiliations:** 1 Bradford Institute for Health Research, Bradford Teaching Hospitals NHS Trust, Bradford, BD9 6RJ UK; 2 School of Clinical & Applied Sciences, Leeds Beckett University, Leeds, LS1 3HE UK; 3 Department of Health Sciences, University of York, York, YO10 5DD UK; 4 Leeds Institute of Clinical Trials Research, University of Leeds, Leeds, LS2 9JT UK

**Keywords:** Ethnicity, Obesity, Food security, Diet

## Abstract

**Background:**

Food insecurity has been associated with dietary intake and weight status in UK adults and children although results have been mixed and ethnicity has not been explored. We aimed to compare prevalence and trajectories of weight and dietary intakes among food secure and insecure White British and Pakistani-origin families.

**Methods:**

At 12 months postpartum, mothers in the Born in Bradford cohort completed a questionnaire on food security status and a food frequency questionnaire (FFQ) assessing their child’s intake in the previous month; at 18 months postpartum, mothers completed a short-form FFQ assessing dietary intake in the previous 12 months. Weights and heights of mothers and infants were assessed at 12-, 24-, and 36-months postpartum, with an additional measurement of children taken at 4–5 years. Associations between food security status and dietary intakes were assessed using Wilcoxon-Mann-Whitney for continuous variables and χ^2^ or Fisher’s exact tests for categorical variables. Quantile and logistic regression were used to determine dietary intakes adjusting for mother’s age. Linear mixed effects models were used to assess longitudinal changes in body mass index (BMI) in mothers and BMI z-scores in children.

**Results:**

At 12 months postpartum, White British mothers reported more food insecurity than Pakistani-origin mothers (11% vs 7%; *p* < 0.01) and more food insecure mothers were overweight. Between 12 and 36 months postpartum, BMI increased more among food insecure Pakistani-origin mothers (β = 0.77 units, [95% Confidence Interval [CI]: 0.40, 1.10]) than food secure (β = 0.44 units, 95% CI: 0.33, 0.55). This was also found in Pakistani-origin children (BMI z-score: food insecure β = 0.40 units, 95% CI: 0.22, 0.59; food secure β = 0.25 units, 95% CI: 0.20, 0.29). No significant increases in BMI were observed for food secure or insecure White British mothers while BMI z-score increased by 0.17 (95% CI: 0.13, 0.21) for food secure White British children. Food insecure mothers and children had dietary intakes of poorer quality, with fewer vegetables and higher consumption of sugar-sweetened drinks.

**Conclusions:**

Food security status is associated with body weight and dietary intakes differentially by ethnicity. These are important considerations for developing targeted interventions.

**Electronic supplementary material:**

The online version of this article (10.1186/s12937-018-0349-7) contains supplementary material, which is available to authorized users.

## Background

Food security is defined as having ‘access by all people at all times to enough food for an active, healthy life’ and takes several forms: quantity (access to enough food), quality (it is nutritionally adequate), and safety (food is safe and was obtained through socially acceptable means without resorting to coping strategies such as emergency food aid) [1, 2]. Food insecurity can therefore be defined as uncertainty surrounding food quality or quantity and has been increasing in Europe since the 2008 recession [3]. Despite the United Kingdom (UK) being one of the largest economies in the world, 10% of the population aged 15 years and older in 2014 have been reported to be food insecure and almost 20% of children aged 15 years and younger were reported to be living with a food insecure individual [2, 4].

The consequences of food insecurity range from anxiety about being able to provide a balanced meal, to worrying that food will run out, to skipping meals. These can lead to not only reductions in diet quality, which is compounded by the cheap and palatable nature of highly processed food, but also reductions in quantity which could lead to frank hunger [2, 5–8]. The level of food insecurity experienced can therefore have wide-ranging impacts and has been associated with poor mental health, difficulty managing chronic diseases, and child behaviour problems [9–16].

A relationship between food insecurity and body weight in women has been observed in previous studies, with increased prevalence of both underweight and overweight [14, 17, 18]. This relationship is less clear in children [12, 14, 19]. In the UK, two studies have assessed how food insecurity may impact dietary intake and body mass: among food insecure women, a reduction in dietary diversity was reported and, for children aged 3 years, there were no differences in body mass index (BMI) but food insecure children consumed more processed meat, crisps, and soft drinks, but fewer vegetables [17, 20]. However, these studies were unable to explore the role of ethnicity. Previous work in our study population have shown ethnic differences in food security status [10], dietary intakes in children [21], and availability of foods and beverages in the home [22]. While studies have indicated that food insecurity may be more prevalent among certain racial and ethnic groups [23, 24], to our knowledge, there are no studies in the UK on whether food insecurity may differentially influence BMI status and dietary intakes by ethnic group. Most studies on the impact of food insecurity on health in high-income countries come from the United States [25], Canada [26], and Australia [27], with few studies from the UK [2, 17, 28, 29].

In a multi-cultural and urban deprived city, this study examined the association of food security status with dietary intake, prevalence of overweight and obesity, and longitudinal BMI and BMI z-score trajectories of White British and Pakistani-origin mothers and children.

## Methods

### Study population

The Born in Bradford (BiB) study is a longitudinal birth cohort which aims to examine the impact of environmental, psychological, and genetic factors on the health and well-being of mothers and children [30]. Bradford is a city in the north of England with high levels of ethnic diversity and socioeconomic deprivation, where approximately half of all BiB births are of Pakistani-origin. The BiB cohort recruited 12,453 women between 2007 and 2010 while they were attending the Bradford Royal Infirmary for universal oral glucose tolerance testing at 26–28 weeks gestation. Women self-completed a semi-structured questionnaire on socio-demographic characteristics, health and lifestyle behaviour, and consented to routine linkage of mother and child data [31]. Non-English speaking mothers were aided by the help of multi-lingual research assistants.

A sub-sample of the BiB cohort, the Born in Bradford 1000 longitudinal study (BiB1000, *n* = 1735), was recruited between August 2008 and March 2009 to obtain more detailed information on diet, anthropometry, social, behavioural, and environmental factors that were hypothesised to be associated with the development of obesity [32]. All women recruited to the BiB birth cohort during this period were invited to take part in BiB1000. Follow-up of the sub-sample occurred at 6-, 12-, 18-, 24-, and 36-months of child’s age, with dietary data collected at 12 and 18 months. In this study, we utilised anthropometric data collected at baseline, 12-, 24-, and 36-months. Ethical approval was granted by the Bradford Research Ethics Committee (ref: 07/H1302/112).

### Food security status

The validated United States Department of Agriculture (USDA) 18-item food security questionnaire was self-completed by English-speaking mothers during the 12-month BiB1000 follow-up assessment. Non-English speaking mothers were aided by multi-lingual research assistants. This questionnaire assesses quantitative and qualitative aspects of food intake and supply for the previous 12 months including: anxiety over inadequate food supply or budget, perceived inadequacy in quality or quantity of foods, and reduced food intake or perceived hunger in the adults or children [1]. Two categories of food security status were defined: food secure, where households reported little or no evidence of food insecurity, and food insecure, where households reported evidence of difficulty in managing access and quality of food intake. Answers were coded as ‘0’ or ‘1’ and summed to obtain a final score; scores < 3 indicated food security, while scores ≥3 indicated food insecurity [1]. Missing responses for items within the questionnaire were coded to ‘0’. Four women were excluded due to non-completion of the entire questionnaire, with ethnicity and food security data available for *n* = 1105 women.

### Dietary intake

At the 18-month assessment, mothers self-completed the validated short-form food frequency questionnaire (SFFFQ) [33], which assessed the frequency of consumption of foods in the previous 12 months. This tool was modified to include foods commonly consumed in the local community such as culturally-appropriate snacks and sweets and take-away foods so that the final SFFFQ included 32 items. Foods were grouped based on energy density and product type to create 11 food groups: fruits, vegetables, potatoes (including fried, mashed), chips (including oven and fried), rice and breads, sweets and cakes, savoury snacks, fast food (including pasties, takeaway, kebabs), natural fruit juice, sugar-sweetened drinks including squash, and low-sugar drinks including low-sugar squash (Additional file 1). Reported maternal dietary intake data were converted into portions per day: rarely or never (0 portions/day), < 1/week (0.05 portions/day), 1/week (0.14 portions/day), 2–3 times/week (0.36 portions/day), 4–6 times/week (0.71 portions/day), 1–2 times/day (1.5 portions/day), 3–4 times/day (3.5 portions/day), 5+ times/day (6 portions/day).

The ‘5 A Day’ recommendation by the UK government encourages the consumption of at least 5 portions of fruits and vegetables each day [34]. Participants were categorised as having met the ‘5 A Day’ or not, excluding potatoes/potato products and fruit juice. A separate variable which included one serving of natural fruit juice as counting towards achieving the ‘5 A Day’ was also created.

Child diet was assessed through a validated food frequency questionnaire (FFQ) [35] at the 12-month visit and was collected by a team of dietitian-trained multilingual community research assistants. Frequency of foods consumed in the previous month was reported by mothers. Additional items were included in the FFQ to reflect foods commonly consumed within the multi-ethnic population based on 24-h recalls; the final FFQ included 98 items.

Key indicator food groups were derived from the child FFQ by grouping foods into high and low energy density categories based on their contribution to the development of obesity [21]. Twelve key indicator food groups, plus water, were formed: baby formula milk, commercial savoury baby foods, commercial sweet baby foods, potatoes (including chips and roasted), processed meat products, vegetables (including tinned and salad), fruits (including fresh, tinned, and dried), cakes and sweets (including biscuits and chocolate), crisps and savoury snacks, sugar-sweetened drinks, juice, and low-sugar drinks. Frequency of consumption was converted into portions/week: never (0 portion/week), 1–3 times per month (0.5 portion/week), 1 day/week (1 portion/week), 2 days/week (2 portions/week), 3 days/week (3 portions/week), 4 days/week (4 portions/week), 5 days/week (5 portions/week), 6 days/week (6 portions/week), 7 days/week (7 portions/week). Consumption of formula milk, commercial sweet and savoury baby foods, and sugar-sweetened and low-sugar drinks were categorised as ‘consumer’ (> 0 portion/week) or ‘non-consumer’ (0 portion/week).

### Body weight

Mother’s body mass index (BMI) was calculated as kg/m^2^ from measured weight (kg; Seca 2in1 scales, Harlow Healthcare Ltd., London, UK) at the 12-, 24- and 36-month visits and height (m) from the baseline visit at the time of booking-in. BMI status categories were then derived following convention: underweight (BMI < 18.5), normal weight (18.5 ≤ BMI < 25), overweight (25 ≤ BMI < 30), and obese (BMI ≥ 30).

Child weights and heights were obtained from the BiB1000 assessment at the 12-, 24-, and 36-month study visits as well as from the National Child Measurement Programme dataset (NCMP) collected when children were between the ages of 4–5 years old and obtained through data linkage. Some children had multiple NCMP measurements; the first measurement recorded after the age of 4 years was retained. The LMSgrowth Excel application was used to obtain the child’s standard deviation scores (SDS) for their BMI, resulting in BMI z-scores which accounted for age and sex [36]. These scores were then used to classify children as overweight, including obese (BMI z-score ≥ 1.04; BMI percentile ≥85th percentile of the British 1990 growth reference).

### Sociodemographic characteristics

Baseline questionnaires obtained information on mother’s ethnicity, age (years), marital and cohabitation status (married; cohabiting with partner; partner, not cohabiting; single), education (A-level equivalent or higher; maximum of 5 General Certificate of Secondary Education [GCSE], unknown, or foreign), number of individuals living within the household in addition to the participant, whether the family received means-tested benefits (yes; no), and a question to elicit subjective feelings of poverty, asking how well they felt they were managing financially (struggling financially; not struggling financially).

Post codes were used to categorise homes into quintiles of national Index of Multiple Deprivation (IMD), with lower quintiles indicating higher deprivation. IMD is used as a measure of relative deprivation for small areas in England. These small areas, known as Lower-layer Super Output Areas, were constructed to have similar population sizes in order to equally divide the country to allow comparison and identification of the most deprived areas [37].

### Data analyses

Participant socio-demographics, BMI status, and dietary intakes were calculated by food security status within ethnic group. We only considered differences between the White British and Pakistani-origin ethnic groups as they were the predominant ethnic groups within BiB1000 (38% and 49%, respectively); the remaining ethnic groups would not contribute to meaningful conclusions due to small sample sizes. Descriptive statistics are presented as mean and standard deviation (SD) for continuous measures and percentage (%) for categorical measures. Differences between food security status within ethnic groups were calculated with χ^2^ or Fisher’s exact tests for categorical variables and Wilcoxon-Mann-Whitney for continuous variables.

Dietary data were available for *n* = 1273 and *n* = 963 women had complete data on ethnicity, food security, and dietary intake. For maternal dietary intakes, fruits and vegetables are reported as portions/day and the remaining foods are reported as portions/week by multiplying portions/day by 7. Among children with ethnicity and food security information, data were available in a range *n* = 1101 to *n* = 1108 for frequency of consumption, and *n* = 604 to *n* = 1106 for consumer/non-consumer data. For children, fruits, vegetables, cakes and sweets, and water intakes are reported as portions/day by dividing their respective portion/week by 7. Portions/week or per day for each food group were summed and reported as median (interquartile range [IQR]) of intake per week or per day as data were right-skewed. Unadjusted differences between food insecurity categories by ethnicity were calculated using Wilcoxon-Mann-Whitney tests for continuous variables, and χ^2^ or Fisher’s exact tests for categorical variables. We used multivariable quantile regression to report median intakes controlling for mother’s age. Reported β coefficients are interpreted as: food secure individuals consuming a median β portions/day or portions/week more or less than food insecure individuals, controlling for mother’s age. Multivariable logistic regression was used for binary response variables (consumer vs. not consumer) to report odds of consuming sweet and savoury commercial baby foods, baby formula milk, and sugar-sweetened and low-sugar drinks for food secure compared to food insecure children. We used linear mixed effects regression models to explore BMI and BMI z-score trajectories by food security status and ethnicity in a subset of children (*n* = 410) and mothers (*n* = 677) who had complete weights and heights at study visits. In these models, time of measurement was set as the fixed effect and individual as random effect, and BMI and BMI z-score as the continuous outcomes for mothers and children, respectively. All statistical analyses were carried out in R version 3.3.3 (R Foundation for Statistical Computing, Vienna, Austria, 2017).

## Results

### Characteristics

The majority of participants were food secure (91%), with Pakistani-origin mothers reporting greater food security than White British mothers (93% vs 89%; *p* < 0.01). Compared to Pakistani-origin food secure mothers, White British food secure mothers were less likely to live in the bottom 40% of areas of deprivation (72% vs 95%) or receive means-tested benefits (Additional file 2). Pakistani-origin food insecure mothers, compared to food secure, were older (30.3 [6.1] vs 27.8 [5.0] years; *p* = 0.01) and reported more individuals living in the household (7.8 [2.1] vs 5.2 [2.2]; *p* = 0.02) (Table. 1). White British food insecure mothers, compared to food secure, were younger (25.1 [5.0] vs 27.8 [6.2] years; *p* < 0.01), more likely to receive means-tested benefits (59% vs 32%; *p* < 0.01), and reported more feelings of subjective poverty (74% vs 27%; *p* < 0.01).

**Table 1:** Characteristics of White British and Pakistani-origin women by food security status

	White British					Pakistani-origin				
	Food secure		Food insecure			Food secure		Food insecure		
	N	Mean (SD)/ %	N	Mean (SD)/ %	*p*-value^*^	N	Mean (SD)/ %	N	Mean (SD)/ %	*p*-value^*^
Mother’s age (years)	426	27.8 (6.2)	54	25.1 (5.0)	0.0006	583	27.8 (5.0)	41	30.3 (6.1)	0.01
Married/cohabitation status										
Married	178	42	15	28	0.1	567	97	38	93	0.1
Cohabiting with partner	158	37	25	46		4	0.7	0	0	
Partner, not cohabiting	33	8	3	6		2	0.3	1	2	
Single	57	13	11	20		11	2	2	5	
Number living in household	55	3.7 (1.1)	10	3.8 (1.5)	0.9	68	5.2 (2.2)	4	7.8 (2.1)	0.02
Mother’s education^a^										
A-level equivalent or higher	163	38	15	28	0.2	228	39	11	27	0.1
Maximum of 5 GCSEs, unknown, foreign, other	263	62	39	72		353	61	30	73	
National IMD										
Quintile 1	207	49	35	65	0.2	461	79	37	90	0.4
Quintile 2	99	23	12	22		91	15.5	4	10	
Quintile 3	77	18	5	9		29	5	0	0	
Quintile 4	25	6	1	2		2	0.3	0	0	
Quintile 5	18	4	1	2		1	0.2	0	0	
Struggling financially										
Yes	117	27	40	74	< 0.0001	180	31	19	46	0.05
No	309	73	14	26		404	69	22	54	
Received means-tested benefits										
Yes	136	32	32	59	0.0001	265	45	22	54	0.3
No	290	68	22	41		319	55	19	46	

IMD, Index of Multiple Deprivation (quintile 1 indicates most deprived; quintile 5 indicates least deprived)

^a^A-level is equivalent to a United States high school diploma

*χ2 or Fisher’s exact test for categorical variables and Wilcoxon-Mann –Whitney for continuous variables between food security status within ethnic group

### Overweight and obesity in mothers

**Fig. 1:**
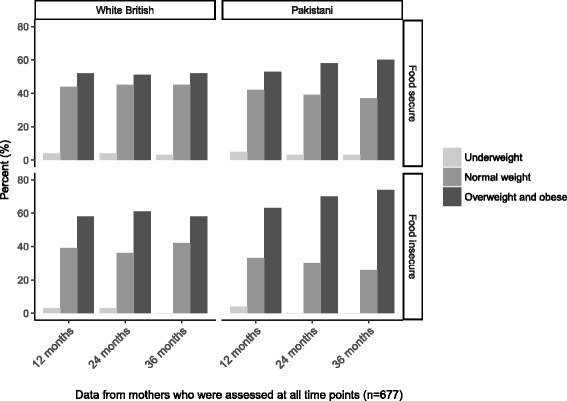
Mothers’ weight status by food security status

Over half of all mothers at 12 months postpartum were overweight or obese (Fig. 1). For Pakistani-origin mothers who participated in all study visits from 12- to 36-months, prevalence of overweight and obesity increased among both those who were food secure (53% to 60%) and food insecure (63% to 74%). However, regression models showed a greater increase in BMI among food insecure Pakistani-origin mothers (β [95% CI]: 0.77 [0.40, 1.10]) compared to those who were food secure (β [95% CI]: 0.44 [0.33, 0.55]) (Table 2). Among White British mothers, overweight and obesity remained at 52% for those who were food secure and 58% for those who were food insecure from 12- to 36-months. Regression models showed that BMI non-significantly increased by 0.14 units for those who were food secure (95% CI: -0.03, 0.31) and 0.18 units for those who were food insecure (95% CI: -0.20, 0.55).

**Table 2:** Change in BMI and BMI z-score for food secure and food insecure White British and Pakistani-origin mothers and children

Mother (BMI)	N	β	(95% CI)
White British			
Food secure	233	0.14	(−0.03, 0.31)
Food insecure	33	0.18	(−0.20, 0.55)
Pakistani-origin			
Food secure	384	0.44	(0.33, 0.55)
Food insecure	27	0.77	(0.40, 1.10)
Child (BMI z-score)			
White British			
Food secure	178	0.17	(0.13, 0.21)
Food insecure	21	0.06	(−0.05, 0.17)
Pakistani-origin			
Food secure	199	0.25	(0.20, 0.29)
Food insecure	12	0.40	(0.22, 0.59)

*BMI* Body Mass Index

Change is from 12–36 months postpartum for mothers and 12 months to 4–5 years old in children

### Overweight and obesity in children

**Fig. 2:**
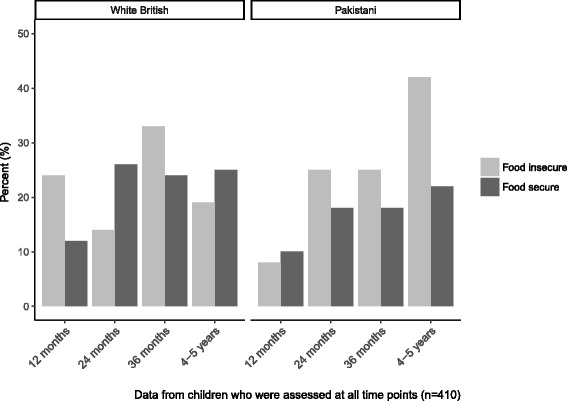
Children’s overweight and obesity by food security status

At 12 months of age, over 10% of all children were overweight or obese. For Pakistani-origin children who attended all study visits and had NCMP data at 4–5 years old, prevalence of overweight and obesity increased among those who were both food secure (10% to 22%) and food insecure (8% to 42%) (Fig. 2). This increase was also found in regression models, where BMI z-score increased by 0.25 units per assessment for Pakistani-origin children who were food secure (95% CI: 0.20, 0.29) and 0.40 units for those who were food insecure (95% CI: 0.22, 0.59) (Table 2). Among White British children, there was a pattern of increasing overweight among the food secure (12% to 25%), but a decrease among the food insecure (24% to 19%); regression modelling showed that BMI z-score increased by 0.17 units (95% CI: 0.13, 0.21) in those who were food secure, and 0.06 (95% CI: -0.05, 0.17) in those who were food insecure.

### Dietary intake in mothers

Pakistani-origin mothers who were food secure consumed fewer portions of fruit compared to those who were food insecure (median [IQR]: 0.36 [0.14, 1.50] vs 0.72 [0.36, 1.50] portions/day; *p* = 0.02) in unadjusted analyses (Additional file 3). This was also found in adjusted analyses, with food secure Pakistani-origin mothers consuming less fruit (β [95% CI]: -0.35 [− 0.55, − 0.35] portions/day) and snacks (β [95% CI]: − 0.91 [− 1.33, − 0.36] portions/week) and more vegetables (β [95% CI]: 0.35 [0.32, 0.35] portions/day) compared to Pakistani-origin mothers who were food insecure (Table 3). Among White British mothers, those who were food secure consumed more vegetables per day compared to those who were food insecure (median [IQR]: 1.07 [0.50, 1.55] vs 0.71 [0.38, 1.24] portions/day; p = 0.02) in unadjusted analyses (Additional file 3). This was also observed in adjusted analyses where food secure White British mothers consumed more vegetables (β [95% CI]: 0.20 [0.001, 0.40] portions/day) compared to food insecure White British mothers (Table 3).

**Table 3:** Adjusted dietary intakes for food secure compared to food insecure White British and Pakistani-origin mothers and children

	White British		Pakistani-origin	
	Ref: food insecure		Ref: food insecure	
	N	β/OR (95% CI)	N	β/OR (95% CI)
Mother’s intake				
Fruits (portions/day)	419	0.09 (−0.02 0.16)	543	−0.35 (−0.55, −0.35)
Vegetables (portions/day)	419	0.20 (0.001, 0.40)	543	0.35 (0.32, 0.35)
Potatoes (portions/week)	419	0.00 (−1.55, 0.00)	543	0.61 (0.12, 0.79)
Chips (portions/week)	419	0.33 (0.12, 0.68)	543	−0.14 (−0.85, 0.29)
Rice and breads (portions/week)	419	0.19 (−0.16, 0.67)	543	−0.24 (−1.12, 0.97)
Sweets and cakes (portions/week)	419	0.35 (−0.38, 1.65)	543	−0.11 (−1.18, 0.65)
Snacks (portions/week)	419	−0.20 (−0.79, 0.86)	543	−0.91 (−1.33, −0.36)
Fast food (portions/week)	419	−0.34 (−0.89, 0.24)	543	0.18 (−0.91, 0.61)
Juices (portions/week)	419	−0.004 (−0.10, 1.52)	543	0.56 (−0.11, 1.35)
Sugar-sweetened beverages, including squash (portions/week)	419	−0.72 (−1.48, 0.50)	543	0.27 (−1.18, 0.77)
Low-sugar beverages, including squash (portions/week)	419	−0.65 (−5.58, 2.17)	543	0.01 (−0.65, 0.59)
Child’s intake				
Baby formula milk*				
Consumer	472	1.11 (0.62, 1.98)	617	1.22 (0.62, 2.55)
Commercial savory baby meals*				
Consumer	274	1.17 (0.37, 3.10)	352	1.11 (0.46, 2.63)
Commercial sweet baby meals*				
Consumer	267	1.53 (0.62, 4.12)	334	0.99 (0.36, 2.49)
Chips, roasts & potato shapes (portions/week)	480	−0.34 (−0.94, 0.05)	623	−0.06 (−0.70, 0.25)
Processed meat products (portions/week)	480	−0.67 (−1.74, 0.32)	624	0.13 (−0.03, 0.19)
Vegetables (inc. tinned & salad) (portions/day)	480	0.14 (−0.11, 0.31)	624	0.45 (0.16, 0.67)
Fruits (inc. fresh, tinned & cooked) (portions/day)	480	0.31 (−0.24, 0.49)	624	0.001 (−0.43, 0.19)
Cakes, biscuits, chocolate and sweets (portions/day)	480	−0.19 (−0.54, 0.11)	624	−0.09 (−0.20, 0.12)
Crisps and savory snacks (portions/week)	479	−0.82 (−1.40, −0.37)	621	−0.25 (−1.32, 1.34)
Sugar-sweetened drinks*				
Consumer	480	0.57 (0.32, 1.01)	622	0.44 (0.21, 0.85)
Pure fruit juices (portions/week)	480	0.07 (−0.04, 0.11)	621	−1.11 (−2.61, 0.12)
Low sugar drinks*				
Consumer	476	0.75 (0.41, 1.38)	622	1.60 (0.76, 3.80)
Water (portions/day)	478	0.002 (−0.01, 0.43)	619	0.01 (−0.01, 0.01)

Adjusted for mother’s age

*Odds ratio (OR) of consuming compared to not consuming for food secure compared to food insecure children

Fewer than 10% of all mothers met the UK recommendation of ‘5 A Day’. There were no differences by food security status (Additional file 4). While not significantly different, Pakistani-origin mothers were more likely to achieve recommendations. When 1 serving of natural fruit juice was included towards reaching the ‘5 A Day’, 13% of all participants were able to meet this goal, with no differences observed between the ethnic groups or by food security status.

### Dietary intakes in children

We did not find differences in dietary intakes between food secure and insecure Pakistani-origin children in unadjusted analyses (Additional file 3). In adjusted analyses, Pakistani-origin children who were food secure consumed more vegetables (β [95% CI]: 0.45 [0.16, 0.67] portions/day) and were less likely to consume sugar-sweetened beverages (OR [95% CI]: 0.44 [0.21, 0.85]) (Table 3). In unadjusted analyses, White British food secure children consumed less processed meat products (median [IQR]: 2.00 [0.50, 4.00] vs 3.00 [1.12, 5.00] portions/week; *p* = 0.01), cakes, biscuits, chocolate and sweets (median [IQR]: 0.71 [0.36, 1.14] vs 0.86 [0.45, 1.66] portions/day; *p* = 0.02), crisps and savoury snacks (median [IQR]: 2.00 [0.00, 3.00] vs 2.00 [1.00, 4.00] portions/week; *p* = 0.002), and sugar-sweetened beverages (39% vs 56% consumers, *p* = 0.02) compared to White British children who were food insecure. In adjusted analyses, White British children who were food secure consumed fewer portions of crisps and savoury snacks (β [95% CI]: -0.82 [-1.40, -0.37] portions/week).

## Discussion

In this exploration of food security status in relation to ethnicity, weight, and dietary intakes in mothers and children, we found dietary intakes and prevalence and rate of overweight and obesity differed by food security status and ethnicity. Overweight and obesity were found to be greater among food insecure mothers and longitudinal analyses confirmed that, among all Pakistani-origin mothers, BMI increased between 12- and 36-months postpartum with greater increases observed among the food insecure. Similar patterns were observed in Pakistani-origin children and in food secure White British children between 12 months and 4–5 years of age. While BMI and BMI z-score also increased over study visits among White British mothers and their food insecure children, these results were not statistically significant.

At 12 months postpartum, prevalence of overweight among food insecure mothers was higher than the UK prevalence of 58% in women [38]. The higher prevalence of overweight and obesity among the food insecure, compared to the food secure, has been observed in other studies [14, 18]. Some studies have also found that the relationship between BMI and food insecurity exhibits a U-shaped curve [39]. When assessing cross-sectionally among all women at 12 months when food security was measured, we observed more underweight among food insecure, compared to food secure, mothers. This was found among White British, but not Pakistani-origin, mothers and may be due to the level of food insecurity experienced; when we further categorised mothers who were food insecure into ‘food insecure without hunger’ or ‘food insecure with hunger’, more White British mothers were categorised as being ‘food insecure with hunger’. As individuals cope with economic restrictions by shifting food quality, BMI may increase as a result of consuming lower-cost but higher-energy foods; a decrease in BMI could result from worsening food insecurity with a shift towards a very-low food security status where quantity of foods are likely to be affected [8, 18].

At 4–5 years old, prevalence of overweight among food insecure Pakistani-origin and food secure White British children was higher than the 22% prevalence reported by the NCMP [40]. In children, studies of food insecurity and weight have reported mixed findings. Some have found positive associations between food insecurity and body weight [16, 41, 42] while others have found none [17, 43], or even negative associations [44, 45]. Our results suggest that the relationship between food insecurity and child’s overweight may differ by ethnicity. This is consistent with results from Alaimo et al. 2001 who found an increased prevalence of overweight by food security status only among non-Hispanic White children in the USA.

We observed a decrease in the prevalence of overweight White British children who were food insecure from the 12-month to 4–5 year assessment. This may be due to a strain on family resources, as White British food insecure mothers reported struggling financially more than their Pakistani-origin counterparts and were more likely to receive means-tested benefits. This could lead to experiencing greater food insecurity, affecting the quantity of food consumed, and may be reflected in their dietary patterns through higher consumption of low-cost, energy-dense foods, such as processed meat products, sweet and savoury snacks, and sugar-sweetened drinks, compared to their food secure counterparts in unadjusted analyses [8]. The monetary and psychologically stressful nature of food insecurity may not only affect availability and consumption of certain foods, but changes in physiological responses, such as levels of cortisol, which may influence appetite and preferences for ‘comfort foods’ that are higher in fat and sugar [46]. However, even with the physiological drive and comparative abundance of energy-dense foods, the relative size and quantity of these foods may be affected.

We did not observe similar findings among Pakistani-origin children. Pakistani-origin families have potentially stronger social networks, such as extended families, which provide social, emotional, or financial support blunting the potential detrimental effects of deprivation. This is suggested through the ‘ethnic density hypothesis’, where health benefits could be derived by ethnic minorities living in high-density areas of their own ethnic groups [6, 21, 47, 48]. These social and financial support networks may also help explain the lower prevalence of food insecurity reported among Pakistani-origin mothers.

We found that food security status was associated with dietary intakes for both Pakistani-origin and White British children with ‘healthier’ consumption among food secure children, such as consuming fewer sugar-sweetened beverages and savoury snacks and more vegetables. Food insecure children may be ‘substituting’ higher nutrient-dense/lower energy-dense foods for more lower nutrient-dense/higher energy-dense foods, which are often cheaper when considered on a per-calorie basis, as well as more palatable [8, 9]. Our findings showing that food secure children consumed more ‘healthy’ foods and less ‘unhealthy’ foods are similar to those reported by Pilgrim et al. (2017). Among 3 year old children in the UK, those living in food secure households consumed more wholemeal bread, yoghurt, and vegetables, and less white bread, processed meat, added sugars, crisps, and soft drinks [17].

A previous study in our population also found greater availability of sugar-sweetened beverages in Pakistani-origin homes [22]. It has been hypothesised that consumption of sugar-sweetened beverages in children is associated with obesity, which may account for the higher prevalence of overweight and obesity observed among our Pakistani-origin mothers and children and particularly among those who were food insecure [49]. This is supported by randomised-controlled trials of sugar-sweetened beverages reduction or substitution with non-sugar beverages that resulted in beneficial changes in body weight or BMI z-score [50–52]. Other studies have found an increase in snacking behaviours associated with those who consume sugar-sweetened beverages, resulting in increased caloric intake [53]. We found greater snacking among food insecure Pakistani-origin mothers, potentially contributing to the steadily increasing BMI observed.

Interestingly, while vegetable consumption was higher among both food secure Pakistani-origin and White British mothers, fruit consumption was higher among food insecure Pakistani-origin mothers compared to those who were food insecure. This could be due to a combination of cost coupled with cultural norms, such as maintaining a traditional diet [54]. Studies have found that fruit and vegetable prices are often lower in deprived areas or areas with high ethnic density, and consumption of fruits and vegetables is frequently higher among minority groups [55–57]. However, fewer than 15% of mothers met the recommended ‘5 A Day’, lower than the reported 28% of women in England who met the guidelines [40].

Nine percent of mothers in our population were categorised as being food insecure; most of our mothers were considered to have marginal food insecurity and could be categorised as being ‘food insecure without hunger’, while nine White British and one Pakistani-origin mother had very low food security and could be categorised as being ‘food insecure with hunger’. This prevalence is similar to that reported by the United Nations Food and Agriculture Organisation (FAO; 10%) but the true number may be higher, as the FAO did not capture less severe experiences of food insecurity such as anxiety around food quality or whether children were affected. Comparison of results between studies may depend on the method by which food insecurity is captured; for example, only individual personal-level, but not household child-level, food insecurity was associated with obesity in a study of children aged 6–11 years [43]. Additionally, some studies used questions that more closely estimate food insufficiency, which would be considered very low food security, whereas other studies utilised part or all of the USDA food security scale. Even in instances of marginal food insecurity where there has not been a significant reduction in food quantity, similar health risks and sociodemographic and psychological patterns have been observed, exemplifying the lack of a clear cut-point where food insecurity impacts health and wellbeing [58]. In our population, food insecurity appeared to mainly affect adults, as only six mothers reported cutting the size of their child’s meal, only five reported that their children were ever hungry, and only one reported that their child skipped a meal.

Limitations of our study include the relatively small sample size of individuals who were food insecure. We categorised food security status into two groups due to sample size concerns, though it has been suggested that marginal food security without hunger is its own risk category [58]. We were also only able to assess food insecurity at a single point in time, though individuals and households can cycle in and out of food security; therefore, this may impact estimation of prevalence as well as its relationship with dietary intake. For questionnaires that were incomplete, we imputed null values and therefore our estimate of food insecurity is conservative. Additionally, we assessed household food security status, which captures status for all children living in the household, though status may differ between children within a household. Our food insecurity measure was developed for use in the United States and may not be the best measure to use within diverse ethnic groups in the UK. Various measures have been used by others, which make direct comparisons of prevalence more difficult [2, 17, 29]. Children’s overweight and obesity was determined using the British 1990, rather than the World Health Organization 2006 growth standards, which could have implications for direct comparison with international studies. We also did not control for multiple comparisons as this was an exploratory analysis, which increases the likelihood of false significant findings. However, our study is one of the first to examine the association of food security status with dietary intake and obesity in the UK, as well as the first to assess differences by ethnicity. We were also able to examine the trend in overweight and obesity prevalence and BMI and BMI z-score trajectory in a subset of our population with multiple assessments over time. Data linkage with the NCMP allowed us to capture child’s weights and heights beyond cohort assessments, and continued follow-up of cohort members will provide opportunities to further examine how dietary intakes are shaped by social, economic, and environmental conditions.

## Conclusion

This study characterised the association of food security status on weight and dietary intakes and suggests that ethnicity, potentially through cultural norms and community support, may lead to differences in how food insecurity is experienced. This indicates that policy measures to assess and support food insecure families need to consider which groups may be more at risk in order to develop and appropriately target interventions. Further research is needed to determine how families may move in and out of food security status and how this may affect child and maternal health over time.

## Additional files

Additional file 1: Groupings of 32 food items from the SFFFQ at 18 months into 11 food groups. (DOCX 19 kb)

Additional file 2: Comparison of White British and Pakistani-origin characteristics by food security status. (DOCX 19 kb)

Additional file 3: Unadjusted dietary intakes for White British and Pakistani-origin mothers and children by food security status. (DOCX 25 kb)

Additional file 4: Proportion of White British and Pakistani-origin mothers who meet the recommended ‘5 A Day’ for fruit and vegetable intake. (DOC 34 kb)

## Abbreviations

BiB: Born in Bradford
BMI: Body Mass Index
CI: Confidence Interval
FAO: Food and Agriculture Organization
FFQ: Food Frequency Questionnaire
GCSE: General Certificate of Secondary Education
IMD: Index of Multiple Deprivation
IQR: Interquartile Range
NCMP: National Child Measurement Programme
SD: Standard Deviation
SFFQ: Short Form Food Frequency Questionnaire
UK: United Kingdom
USDA: United States Department of Agriculture
