# Supplementary material for: Association of food security status with overweight and dietary intake: exploration of White British and Pakistani-origin families in the Born in Bradford cohort
Source: Nutr J. 2018 Apr 24;17:48. doi: 10.1186/s12937-018-0349-7 (PMC5916586; doi:10.1186/s12937-018-0349-7)
Supplement: Supplementary file 1 — Groupings of 32 food items from the SFFFQ at 18 months into 11 food groups. (DOCX 19 kb) [file 12937_2018_349_MOESM1_ESM.docx]

| **Food group** | **Food items** |
| --- | --- |
| Fruits | Fruits |
| Vegetables | Vegetables, salad |
| Potatoes | Boiled, mashed, jacket, fried, roasted potatoes |
| Chips | Oven-cooked, fried chips |
| Rice and breads | Fried rice, biryani, boiled rice, chapattis, naan, parathas, puris |
| Sweets and cakes | Biscuits, cakes, pastries, Indian sweets |
| Snacks | Crisps and savory snacks, Chevda, Bombay mix, samosas, pakoras, spring rolls, sausage rolls |
| Fast food | Meat and vegetarian pies, pasties, pizza, quiche, flan, meals from chip shops, beef and vegetable burgers, fried chicken, kebabs, takeaway |
| Juices | Natural fruit juice, mango juice |
| Sugar-sweetened beverages, including squash | Sugar-sweetened carbonated beverages, fruit drinks, squash |
| Low-sugar beverages including squash | Sugar-free carbonated beverages, fruit drinks, squash |
