# Supplementary material for: Association of food security status with overweight and dietary intake: exploration of White British and Pakistani-origin families in the Born in Bradford cohort
Source: Nutr J. 2018 Apr 24;17:48. doi: 10.1186/s12937-018-0349-7 (PMC5916586; doi:10.1186/s12937-018-0349-7)
Supplement: Supplementary file 2 — Comparison of White British and Pakistani-origin characteristics by food security status. (DOCX 19 kb) [file 12937_2018_349_MOESM2_ESM.docx]

|  | **Food secure** | | | |  |  | **Food insecure** | | | |  |
| --- | --- | --- | --- | --- | --- | --- | --- | --- | --- | --- | --- |
|  | White British | | Pakistani-origin | |  |  | White British | | Pakistani-origin | |  |
|  | N | Mean (SD)/ % | N | Mean (SD)/ % | *p*-value^*^ |  | N | Mean (SD)/ % | N | Mean (SD)/ % | *p*-value^*^ |
| **Mother's age (years)** | 426 | 27.8 (6.2) | 583 | 27.8 (5.0) | 0.9 |  | 54 | 25.1 (5.0) | 41 | 30.3 (6.1) | <0.0001 |
| **Married/Cohabitation** |  |  |  |  |  |  |  |  |  |  |  |
| Married | 178 | 42 | 567 | 97 | <0.0001 |  | 15 | 28 | 38 | 93 | <0.0001 |
| Cohabiting with partner | 158 | 37 | 4 | 0.7 |  |  | 25 | 46 | 0 | 0 |  |
| Partner, not cohabiting | 33 | 8 | 2 | 0.3 |  |  | 3 | 6 | 1 | 2 |  |
| Single | 57 | 13 | 11 | 2 |  |  | 11 | 20 | 2 | 5 |  |
| **Number living in household** | 55 | 3.7 (1.1) | 68 | 5.2 (2.2) | <0.0001 |  | 10 | 3.8 (1.5) | 4 | 7.8 (2.1) | 0.02 |
| **Mother's education**ⱡ |  |  |  |  |  |  |  |  |  |  |  |
| A-level equivalent or higher | 163 | 38 | 228 | 39 | 0.8 |  | 15 | 28 | 11 | 27 | 1 |
| Maximum of 5 GCSEs, unknown, foreign, other | 263 | 62 | 353 | 61 |  |  | 39 | 72 | 30 | 73 |  |
| **National IMD** |  |  |  |  |  |  |  |  |  |  |  |
| Quintile 1 | 207 | 49 | 461 | 79 | <0.0001 |  | 35 | 65 | 37 | 90 | 0.5 |
| Quintile 2 | 99 | 23 | 91 | 15.5 |  |  | 12 | 22 | 4 | 10 |  |
| Quintile 3 | 77 | 18 | 29 | 5 |  |  | 5 | 9 | 0 | 0 |  |
| Quintile 4 | 25 | 6 | 2 | 0.3 |  |  | 1 | 2 | 0 | 0 |  |
| Quintile 5 | 18 | 4 | 1 | 0.2 |  |  | 1 | 2 | 0 | 0 |  |
| **Struggling financially** |  |  |  |  |  |  |  |  |  |  |  |
| Yes | 117 | 27 | 180 | 31 | 0.3 |  | 40 | 74 | 19 | 46 | 0.01 |
| No | 309 | 73 | 404 | 69 |  |  | 14 | 26 | 22 | 54 |  |
| **Received means-tested benefits** |  |  |  |  |  |  |  |  |  |  |  |
| Yes | 136 | 32 | 265 | 45 | <0.0001 |  | 32 | 59 | 22 | 54 | 0.7 |
| No | 290 | 68 | 319 | 55 |  |  | 22 | 41 | 19 | 46 |  |
| IMD, Index of Multiple Deprivation (quintile 1 indicates most deprived; quintile 5 indicates least deprived)  ⱡA-level is equivalent to a United States high school diploma  ^*^χ2 or Fisher's exact test for categorical variables or Wilcoxon-Mann-Whitney for continuous variables between ethnic groups within food security status | | | | | | | | | |  |  |
