# Supplementary material for: Association of food security status with overweight and dietary intake: exploration of White British and Pakistani-origin families in the Born in Bradford cohort
Source: Nutr J. 2018 Apr 24;17:48. doi: 10.1186/s12937-018-0349-7 (PMC5916586; doi:10.1186/s12937-018-0349-7)
Supplement: Supplementary file 3 — Unadjusted dietary intakes for White British and Pakistani-origin mothers and children by food security status. (DOCX 25 kb) [file 12937_2018_349_MOESM3_ESM.docx]

|  | | | | | | | | | | | | | |
| --- | --- | --- | --- | --- | --- | --- | --- | --- | --- | --- | --- | --- | --- |
|  | **Food secure** | | | |  | **Food insecure** | | | |  | |  |  |
|  | White British | | Pakistani-origin | |  | White British | | Pakistani-origin | |  | |  |  |
|  | N | Median (IQR)/ % | N | Median (IQR)/ % | *p*-value^*^ | N | Median (IQR)/ % | N | Median (IQR)/ % | | *p*-value^*^ | *p*-value^†^ | *p*-value^‡^ |
| **Mother's intake** |  |  |  |  |  |  |  |  |  | |  |  |  |
| Fruits (portions/day) | 372 | 0.36 (0.14, 1.50) | 506 | 0.36 (0.14, 1.50) | 0.6 | 47 | 0.36 (0.14, 0.71) | 38 | 0.72 (0.36, 1.50) | | 0.003 | 0.08 | 0.02 |
| Vegetables (portions/day) | 372 | 1.07 (0.50, 1.55) | 506 | 1.07 (0.50, 1.86) | 0.09 | 47 | 0.71 (0.38, 1.24) | 38 | 0.72 (0.50, 1.81) | | 0.5 | 0.02 | 0.1 |
| Potatoes (portions/week) | 372 | 3.50 (2.52, 5.04) | 506 | 1.33 (0.35, 2.87) | <0.0001 | 47 | 3.50 (2.52, 5.32) | 38 | 0.98 (0, 2.52) | | <0.0001 | 0.3 | 0.2 |
| Chips (portions/week) | 372 | 0.98 (0.35, 2.52) | 506 | 1.33 (0.98, 2.52) | 0.01 | 47 | 0.98 (0.52, 2.52) | 38 | 1.16 (0.98, 2.52) | | 0.7 | 0.6 | 0.9 |
| Rice and breads (portions/week) | 372 | 1.33 (0.35, 2.52) | 506 | 11.82 (7.91, 14) | <0.0001 | 47 | 0.98 (0.35, 2.17) | 38 | 11.87 (7.79, 14) | | <0.0001 | 0.2 | 1 |
| Sweets and cakes (portions/week) | 372 | 2.87 (0.98, 5.04) | 506 | 3.50 (0.98, 10.50) | 0.004 | 47 | 2.52 (0.98, 3.50) | 38 | 3.50 (1.33, 10.76) | | 0.05 | 0.1 | 0.9 |
| Snacks (portions/week) | 372 | 2.52 (0.98, 3.50) | 506 | 2.52 (0.98, 3.50) | 0.5 | 47 | 2.52 (0.98, 3.68) | 38 | 2.69 (1.33, 5.04) | | 0.5 | 0.4 | 0.2 |
| Fast food (portions/week) | 372 | 2.3 (1.3, 3.7) | 506 | 2.3 (1.0, 4.6) | 0.3 | 47 | 2.94 (1.54, 4.24) | 38 | 2.03 (0.77, 4.24) | | 0.3 | 0.2 | 0.4 |
| Juices (portions/week) | 372 | 2.52 (0.35, 4.97) | 506 | 2.52 (0.35, 5.04) | 0.5 | 47 | 2.52 (0.98, 5.0) | 38 | 2.24 (0.70, 3.50) | | 0.8 | 0.6 | 0.7 |
| Sugar-sweetened beverages, including squash (portions/week) | 372 | 0.35 (0, 2.87) | 506 | 1.33 (0, 4.97) | 0.0001 | 47 | 0.98 (0, 4.24) | 38 | 0.70 (0.08, 3.34) | | 0.9 | 0.1 | 0.7 |
| Low-sugar beverages, including squash (portions/week) | 372 | 4.97 (0.91, 10.50) | 506 | 0.98 (0, 3.50) | <0.0001 | 47 | 4.97 (0.98, 10.68) | 38 | 0.35 (0, 2.52) | | 0.0001 | 0.5 | 0.5 |
| **Child's intake** |  |  |  |  |  |  |  |  |  | |  |  |  |
| **Baby formula milk** |  |  |  |  | <0.0001 |  |  |  |  | | 0.1 | 0.8 | 0.6 |
| Consumer | 204 | 49 | 197 | 34 |  | 25 | 46 | 12 | 29 | |  |  |  |
| Non-consumer | 214 | 51 | 380 | 66 |  | 29 | 54 | 29 | 71 | |  |  |  |
| **Commercial savory baby meals** |  |  |  |  | <0.0001 |  |  |  |  | | 0.1 | 0.8 | 0.8 |
| Consumer | 203 | 81 | 195 | 59 |  | 19 | 79 | 13 | 57 | |  |  |  |
| Non-consumer | 47 | 19 | 134 | 41 |  | 5 | 21 | 10 | 43 | |  |  |  |
| **Commercial sweet baby meals** |  |  |  |  | <0.0001 |  |  |  |  | | 0.03 | 0.5 | 1 |
| Consumer | 97 | 40 | 205 | 65 |  | 7 | 30 | 14 | 67 | |  |  |  |
| Non-consumer | 147 | 60 | 108 | 35 |  | 16 | 70 | 7 | 33 | |  |  |  |
| **Chips, roasts & potato shapes (portion/week)** | 426 | 0.50 (0.00, 2.00) | 583 | 1.00 (0.50, 2.00) | <0.0001 | 54 | 1.00 (0.12, 2.00) | 41 | 1.00 (0.50, 3.00) | | 0.3 | 0.07 | 0.5 |
| **Processed meat products (portion/week)** | 426 | 2.00 (0.50, 4.00) | 584 | 0.00 (0.00, 1.00) | <0.0001 | 54 | 3.00 (1.12, 5.00) | 41 | 0.00 (0.00, 0.50) | | <0.0001 | 0.01 | 0.2 |
| **Vegetables (inc. tinned & salad) (portion/day)** | 426 | 1.64 (1.00, 2.34) | 584 | 1.79 (0.86, 2.71) | 0.3 | 54 | 1.43 (0.95, 2.00) | 41 | 1.29 (0.86, 2.57) | | 0.9 | 0.1 | 0.2 |
| **Fruits (inc. fresh, tinned & cooked) (portion/day)** | 426 | 1.43 (0.80, 2.29) | 584 | 2.00 (1.29, 3.00) | <0.0001 | 54 | 1.11 (0.52, 2.14) | 41 | 2.00 (1.43, 2.86) | | 0.0006 | 0.2 | 0.7 |
| **Cakes, biscuits, chocolate and sweets (portion/day)** | 426 | 0.71 (0.36, 1.14) | 584 | 0.57 (0.21, 1.14) | 0.01 | 54 | 0.86 (0.45, 1.66) | 41 | 0.64 (0.29, 1.14) | | 0.04 | 0.02 | 0.7 |
| **Crisps and savory snacks (portion/week)** | 425 | 2.00 (0.00, 3.00) | 581 | 2.00 (0.05, 4.00) | 0.0004 | 54 | 2.00 (1.00, 4.00) | 41 | 2.00 (0.00, 5.00) | | 0.3 | 0.002 | 0.6 |
| **Sugar-sweetened drinks** |  |  |  |  | <0.0001 |  |  |  |  | | 0.3 | 0.02 | 0.05 |
| Consumer | 165 | 39 | 302 | 52 |  | 30 | 56 | 28 | 68 | |  |  |  |
| Non-consumer | 261 | 61 | 280 | 48 |  | 24 | 44 | 13 | 32 | |  |  |  |
| **Pure fruit juices (portion/week)** | 426 | 0.00 (0.00, 4.00) | 581 | 1.00 (0.00, 7.00) | 0.0001 | 54 | 0.00 (0.00, 1.75) | 41 | 2.00 (0.00, 5.00) | | 0.002 | 0.08 | 0.6 |
| **Low sugar drinks** |  |  |  |  | 1 |  |  |  |  | | 0.07 | 0.2 | 0.3 |
| Consumer | 120 | 28 | 166 | 29 |  | 20 | 37 | 8 | 20 | |  |  |  |
| Non-consumer | 302 | 72 | 416 | 71 |  | 34 | 63 | 33 | 80 | |  |  |  |
| **Water (portions/day)** | 424 | 1.00 (0.43, 1.00) | 579 | 1.00 (1.00, 1.00) | <0.0001 | 54 | 1.00 (0.14, 1.00) | 41 | 1.00 (1.00, 1.00) | | 0.003 | 0.04 | 0.3 |
| IQR, interquartile ranges  ^*^χ2 or Fisher's exact test for categorical variables and Wilcoxon-Mann-Whitney for continuous variables between ethnic groups within food security status | | | | | | | | | | | | |  |
| ^†^Test between food security status for White British women | | | | | |  |  |  |  | |  |  |  |
| ^†^Test between food security status for Pakistani-origin women | | | | | | |  |  |  | |  |  |  |
