# Supplementary material for: Association of food security status with overweight and dietary intake: exploration of White British and Pakistani-origin families in the Born in Bradford cohort
Source: Nutr J. 2018 Apr 24;17:48. doi: 10.1186/s12937-018-0349-7 (PMC5916586; doi:10.1186/s12937-018-0349-7)
Supplement: Supplementary file 4 — Proportion of White British and Pakistani-origin mothers who meet the recommended ‘5 A Day’ for fruit and vegetable intake. (DOC 34 kb) [file 12937_2018_349_MOESM4_ESM.doc]

|  |  | | | | |  | | | | |
| --- | --- | --- | --- | --- | --- | --- | --- | --- | --- | --- |
|  | **Food secure** | | | | | **Food insecure** | | | | |
|  | White British | | Pakistani-origin | |  | White British | | Pakistani-origin | |  |
|  | N | % | N | % | *p-*value* | N | % | N | % | *p-*value* |
| **≥ "5-a-day"** |  |  |  |  |  |  |  |  |  |  |
| Yes | 33 | 9 | 50 | 10 | 0.7 | 3 | 6 | 3 | 8 | 1 |
| No | 339 | 91 | 456 | 90 |  | 44 | 94 | 35 | 92 |  |
| **≥ "5-a-day" inc. juices** |  |  |  |  |  |  |  |  |  |  |
| Yes | 42 | 11 | 71 | 14 | 0.3 | 4 | 8 | 5 | 13 | 0.5 |
| No | 330 | 89 | 435 | 86 |  | 43 | 92 | 33 | 87 |  |

**p-*value from χ^2^ test
